# Supplementary material for: Microstructures at the distal tip of ant chemosensory sensilla
Source: Sci Rep. 2022 Nov 11;12:19328. doi: 10.1038/s41598-022-21507-7 (PMC9652420; doi:10.1038/s41598-022-21507-7)
Supplement: Supplementary file 1 — Supplementary Figures. [file 41598_2022_21507_MOESM1_ESM.docx]

**Supplementary Figure S1. Various sample preparation methods do not alter basiconic tip microstructure.** Images of basiconic sensilla from *C. pennsylvanicus* intermediate worker antennae prepared by either (a) washes with hexanes, acetone, and 95% ethanol, (b) washes with acetone and 95% ethanol only, or (c) no washes.


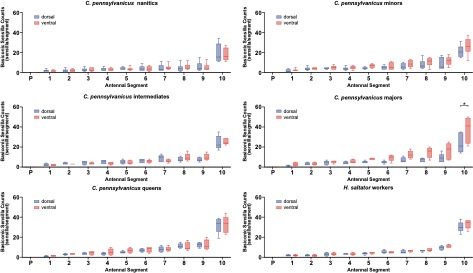


**Supplementary Figure S2. Abundance of Basiconic Sensilla by Antennal Segment (pedicel, P, and funiculus segments 1-10) and Dorsal/Ventral Surface.** In all datasets, the antennal segment had a significant effect on basiconic sensillum abundance (two-way ANOVA, p<0.0001 for all datasets, *n* = 5-6). For minor and major workers of *C. pennsylvanicus*, dorsal/ventral surface had a significant effect on sensillum counts (two-way ANOVA, p=0.0034 for minors and p<0.0001 for majors). Bonferroni’s multiple comparisons test was used for post-hoc comparisons between dorsal/ventral abundance at each segment and p<0.05 is denoted by an asterisk.

**Supplementary Figure S3. Phylogenetic relationships between ant subfamilies used in this study.** Cladogram of ant subfamilies and species numbers created from data in Borowiec et al^1^.

References:

1. Borowiec, M. L., Moreau, C. S. & Rabeling, C. Encyclopedia of Social Insects. 1–18 (2020) doi:10.1007/978-3-319-90306-4_155-1.
